# Rearranging the domain order of a diabody-based IgG-like bispecific antibody enhances its antitumor activity and improves its degradation resistance and pharmacokinetics

**DOI:** 10.4161/mabs.29445

**Published:** 2014-10-30

**Authors:** Ryutaro Asano, Ippei Shimomura, Shota Konno, Akiko Ito, Yosuke Masakari, Ryota Orimo, Shintaro Taki, Kyoko Arai, Hiromi Ogata, Mai Okada, Shozo Furumoto, Masayoshi Onitsuka, Takeshi Omasa, Hiroki Hayashi, Yu Katayose, Michiaki Unno, Toshio Kudo, Mitsuo Umetsu, Izumi Kumagai

**Affiliations:** 1 Department of Biomolecular Engineering; Graduate School of Engineering; Tohoku University; Sendai, Japan; 2 Department of Pharmacology; Tohoku University School of Medicine; Sendai, Japan; 3 Institute of Technology and Science; The University of Tokushima; Tokushima, Japan; 4 Division of Gastroenterological Surgery; Department of Surgery; Graduate School of Medicine; Tohoku University; Sendai, Japan; 5 Cell Resource Center for Biomedical Research; Institute of Development, Aging and Cancer; Tohoku University; Sendai, Japan

**Keywords:** antibody engineering, bispecific diabody, cancer immunotherapy, CD3, effective domain order, epidermal growth factor receptor, IgG-like bispecific antibody

## Abstract

One approach to creating more beneficial therapeutic antibodies is to develop bispecific antibodies (bsAbs), particularly IgG-like formats with tetravalency, which may provide several advantages such as multivalent binding to each target antigen. Although the effects of configuration and antibody-fragment type on the function of IgG-like bsAbs have been studied, there have been only a few detailed studies of the influence of the variable fragment domain order. Here, we prepared four types of hEx3-scDb-Fc, IgG-like bsAbs, built from a single-chain hEx3-Db (humanized bispecific diabody [bsDb] that targets epidermal growth factor receptor and CD3), to investigate the influence of domain order and fusion manner on the function of a bsDb with an Fc fusion format. Higher cytotoxicities were observed with hEx3-scDb-Fcs with a variable light domain (VL)–variable heavy domain (VH) order (hEx3-scDb-Fc-LHs) compared with a VH–VL order, indicating that differences in the Fc fusion manner do not affect bsDb activity. In addition, flow cytometry suggested that the higher cytotoxicities of hEx3-scDb-Fc-LH may be attributable to structural superiority in cross-linking. Interestingly, enhanced degradation resistance and prolonged in vivo half-life were also observed with hEx3-scDb-Fc-LH. hEx3-scDb-Fc-LH and its IgG2 variant exhibited intense in vivo antitumor effects, suggesting that Fc-mediated effector functions are dispensable for effective anti-tumor activities, which may cause fewer side effects. Our results show that merely rearranging the domain order of IgG-like bsAbs can enhance not only their antitumor activity, but also their degradation resistance and in vivo half-life, and that hEx3-scDb-Fc-LHs are potent candidates for next-generation therapeutic antibodies.

## Abbreviations

ADCC: antibody-dependent cell-mediated cytotoxicity
AUC: area-under-the-curve
bsAb: bispecific antibody
bsDb: bispecific diabody
EGFR: epidermal growth factor receptor
FITC-CD3εγ: fluorescein isothiocyanate-labeled CD3εγ; DVD-Ig^TM^, dual variable domain immunoglobulin
FITC-sEGFR: FITC-labeled sEGFR
Fv: variable fragment
ICR: imprinting control region
MTS: 3-(4, 5-dimethylthiazole-2-yl)-5-(3-carboxymethoxyphenyl)-2-(4-sulfophenyl)-2H-tetrazolium inner salt
PBMCs: peripheral blood mononuclear cells
PBS: phosphate-buffered saline
scDb: single-chain diabody
scFv: single-chain Fv
SDS-PAGE: sodium dodecyl sulfate–polyacrylamide gel electrophoresis
sEGFR: soluble EGFR
SPR: surface plasmon resonance
SUV: standardized uptake value
T-LAK cells: lymphokine-activated killer cells with the T-cell phenotype
taFv: tandem scFv
VH: variable heavy domain
VL: variable light domain

## Introduction

Conventional monoclonal antibodies have been widely used for the treatment of a variety of diseases including cancer; however, there remains substantial medical need for improved therapeutic agents. Therefore, many strategies have been developed to improve the functions of antibodies, with one such important approach being the development of bispecific antibodies (bsAbs) capable of simultaneous binding to two different targets.^1^ Bispecificity can be used to cross-link various immune cells, such as cytotoxic T cells, to cancer cells. Although the potential of this approach has been suggested by numerous studies over the years, the difficulty of producing large amounts of homogenous bsAbs using currently available techniques, such as hybrid hybridomas and chemical cross-linking, has hindered their wider adoption and development as therapeutic reagents.^2^

Recombinant technology can be used to generate small bsAb fragments constructed from two different variable antibody fragments, such as variable fragments (Fvs) and single-chain Fvs (scFvs). These bsAb fragments include diabodies (Dbs),^3^ single-chain diabodies (scDbs),^4^ tandem scFvs (taFvs),^5^ and minibodies (dimeric scDb-CH3 fusion proteins).^6^ Compared with classic bsAbs, bsAb fragments have a more convenient size for rapid tissue penetration and high target retention;^7,8^ however, their rapid blood clearance and monovalency may limit their therapeutic application. Technological advances have also enabled the rebuilding of dissected antibody fragments and bsAb fragments into multivalent and more effective formats. IgG-like bsAbs containing a human Fc region are one of the most attractive formats because they may provide several advantages: multivalent binding to each target antigen, prolonged half-life, purification with protein A, and induction of Fc-mediated effector function such as antibody-dependent cell-mediated cytotoxicity (ADCC).^9,10^

The effects of configuration and antibody fragment type on the function of IgG-like bsAbs have been recently reported; for example, using stable scFvs as building blocks was shown to improve the quality of IgG-like bsAbs built from them.^11,12^ However, there have been only a few detailed studies on the influence of the domain order of the variable fragments on the quality of IgG-like bsAbs. We previously described the construction of a functional humanized bispecific Db (bsDb) that targets epidermal growth factor receptor (EGFR) and CD3 (hEx3-Db),^13^ and reported on the cytotoxic enhancement of hEx3-Db by constructing it in its Fc fusion format, hEx3-scDb-Fc.^14,15^ There are, however, two possible domain orders for each chimeric single-chain component of a bsDb: variable heavy domain (VH)–linker–variable light domain (VL) and VL–linker–VH. Varying the order of the domains of both components means that four kinds of bsDb can be constructed from the same set of Fvs. Several previous reports have suggested that the order of the V domain might affect the function of small bsAb fragments such as bispecific taFv and scDb,^16,17^ and we also recently demonstrated cytotoxic enhancement of hEx3-Db by rearranging the domain order.^18^ In the case of constructing an IgG-like bsAb, two fusion options are available for each bsDb, depending on which chimeric single-chain component of the bsDb is fused to the Fc region. Here, taking into account our previous results with hEx3-Dbs without Fc,^18^ we prepared four types of hEx3-scDb-Fc from the eight possible domain orders to investigate the effects of domain order and fusion manner on the function of these antibodies in their Fc-fusion formats.

Similar to hEx3-Dbs,^18^ hEx3-scDb-Fcs with the VL–VH order (hEx3-scDb-Fc-LHs) inhibited tumor cell growth more effectively than did antibodies with the VH–VL order. Notably, hEx3-scDb-Fc-LHs also exhibited enhanced degradation resistance and prolonged in vivo half-lives. Further, intense in vivo antitumor effects were observed with both hEx3-scDb-Fc-LH and its IgG2 variant, suggesting that ADCC activities are not essential for effective anti-tumor activities. Excessive immune activation mediated by both the cross-linking effect and binding to Fc receptors (FcRs) may result in severe side effects, such as the induction of a cytokine storm. Our results show that an effective domain order of bsDbs is retained after Fc fusion, and that hEx3-scDb-Fc-LH, and especially its IgG2 variant, are attractive candidates for next-generation therapeutic antibodies with potent anti-tumor effects, degradation resistance, long half-lives, and a low risk of side effects.

## Results

### Preparation of hEx3-scDb-Fcs with different domain orders

**Figure 1.**
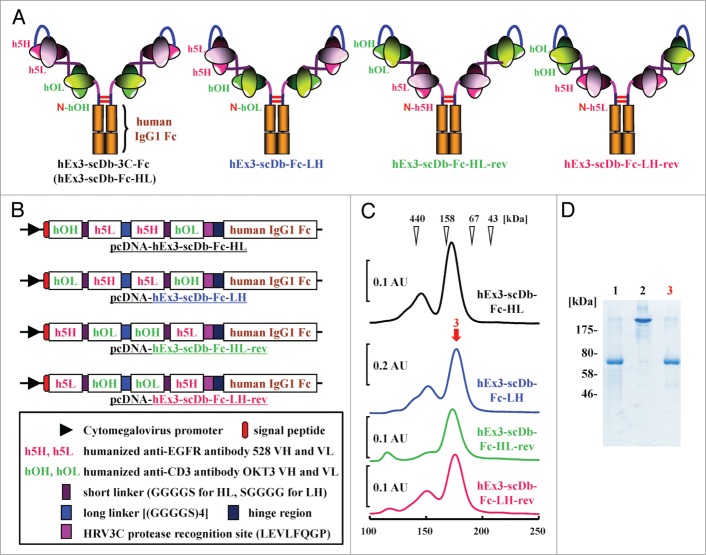
Preparation of hEx3-scDb-Fcs with different domain orders. (**A**) Schematic diagrams of four types of hEx3-scDb-Fc. (**B**) Schematic diagrams of the expression vectors for hEx3-scDb-Fcs. (**C**) Gel filtration of hEx3-scDb-Fcs purified through protein A. AU, absorbance unit. (**D**) SDS-PAGE of each purification step in the preparation of hEx3-scDb-Fc-LH under reducing conditions (lane 1, 3) and non-reducing conditions (lane 2). Lanes 1, 2, after protein A purification; lane 3, peak fraction of gel filtration indicated by the arrow.

To investigate the effects of rearranging the domain order of hEx3-Db on the functions of its Fc fusion format, we prepared four types of hEx3-scDb-Fc (including the original hEx3-scDb-3C-Fc [hEx3-scDb-Fc-HL]), each with a different domain order, as described in **Experimental Procedures**. Schematic diagrams of the four types of hEx3-scDb-Fc and their expression vectors are shown in **Figure 1A and B**, respectively. Protein A chromatography–purified hEx3-scDb-Fcs were applied to gel filtration columns for further purification. Each hEx3-scDb-Fc predominantly formed monomers, which corresponded to the functional scDb-Fc fraction (∼158 kDa); however, small amounts of multimeric forms also appeared on the chromatographs (**Fig. 1C**). For our subsequent analyses, we used the fractionated monomers whose high purity was verified by SDS-PAGE; results for hEx3-scDb-Fc-LH are shown in **Figure 1D** as a representative example. These results show that we successfully prepared the four hEx3-scDb-Fcs with different domain orders.

### Effect of the domain order of scDb on growth inhibition

**Figure 2.**
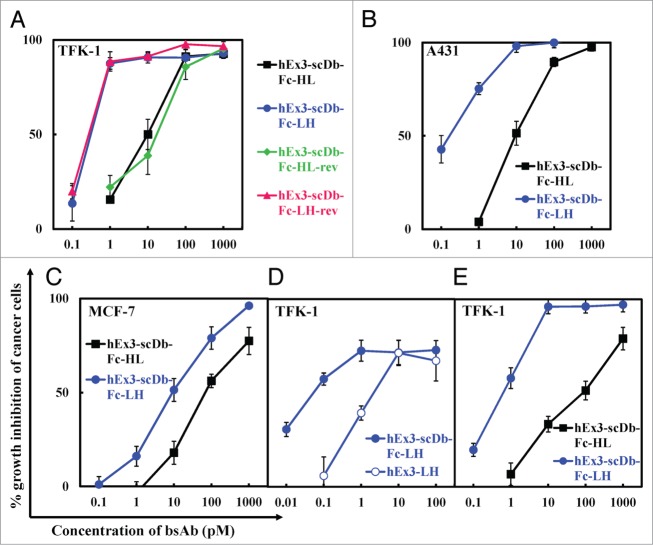
Comparison of growth inhibitory effects of hEx3-scDb-Fcs. hEx3s and T-LAK cells were added to TFK-1 cells (**A, D**), A431 cells (**B**), or MCF-7 cells (**C**) at a ratio of 3:1 (**A, D**) or 2:1 (**B, C**). The indicated hEx3s and PBMCs were added to TFK-1 cells at a ratio of 10:1 (**E**). Data are presented as the mean ± 1 s.d. and are representative of at least two independent experiments.

To evaluate the influence of the domain order of scDb on the inhibition of human carcinoma cell growth, we analyzed the four fractionated hEx3-scDb-Fc monomers by using MTS. Similar to our previous results with hEx3-Dbs,^18^ hEx3-scDb-Fc with the VL–VH order inhibited tumor cell growth more effectively than did hEx3-scDb-Fc-HL in the presence of T-LAK cells (**Fig. 2A**). In contrast, the position of the scDb with respect to the Fc region did not influence the growth inhibition effects; comparable results were observed for hEx3-scDb-Fc-HL and -HL-rev, and for hEx3-scDb-Fc-LH and -LH-rev, respectively. The superiority of hEx3-scDb-Fc-LHs relative to the HL-types was reiterated against A431 cells, which have high EGFR expression (**Fig. 2B**) and also against MCF-7 cells, which have low EGFR expression (**Fig. 2C**). Further, hEx3-scDb-Fc-LH inhibited cancer growth better than did hEx3-LH without the Fc region (**Fig. 2D**), and also more effectively than did hEx3-scDb-Fc-HL when PBMCs were applied as effector cells (**Fig. 2E**). These results indicate that rearranging the domain order of scDbs can enhance the functions of their Fc-fusion formats.

### Influence of the relative position of scDb on the growth inhibition effects of scDb-Fcs

**Figure 3.**
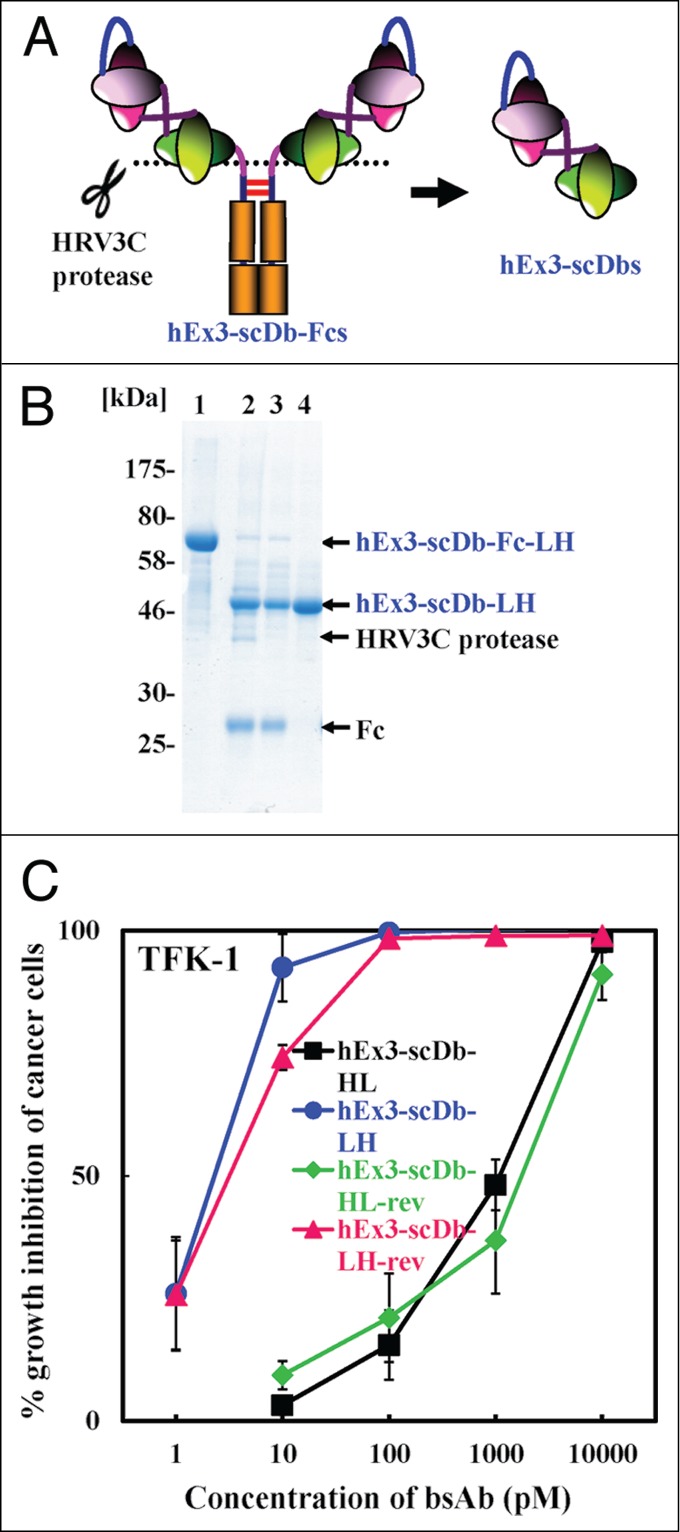
Comparison of growth inhibitory effects of hEx3-scDbs prepared from their Fc-fusion formats. (**A**) Schematic illustration of the preparation of hEx3-scDbs from hEx3-scDb-Fcs. (**B**) Reducing SDS-PAGE of each purification step in the preparation of hEx3-scDb-LH. Lane 1, protein A chromatography–purified hEx3-scDb-Fc-LH; lane 2, after HRV3C protease digestion; lane 3, after removal of HRV3C protease by Glutathione Sepharose 4B chromatography; lane 4, purified hEx3-scDb-LH after removal of the Fc region by protein A chromatography. (**C**) hEx3-scDbs and T-LAK cells were added to TFK-1 cells at a ratio of 3:1. Data are presented as the mean ± 1 s.d. and are representative of at least two independent experiments.

To evaluate the influence of the relative position of scDb against the Fc region on the inhibition of cancer growth in more detail, we digested the Fc region from hEx3-scDb-Fcs by using HRV3C protease to prepare four hEx3-scDbs with different domain orders (**Fig. 3A**) and analyzed the resulting fragments by using MTS. SDS-PAGE analysis of each purification step showed the successful preparation of hEx3-scDbs from their Fc-fusion formats (results for hEx3-scDb-LH are shown in **Figure 3B** as a representative example). Similar to our results with hEx3-scDb-Fcs, both hEx3-scDbs with the VL–VH order inhibited tumor cell growth more effectively than did their HL-order counterparts, and comparable effects were observed among the LH types and also among the HL types (**Fig. 3C**). Therefore, the manner in which scDb is fused to the Fc region and the connecting order of the two chimeric single-chain components of scDb via the peptide linker have no or little effect on the Db's function, indicating that only the domain order of the Db is important for the construction of a highly functional scDb-Fc, at least for hEx3-scDb-Fcs.

### Comparison of binding constants and cross-linking ability

**Figure 4.**
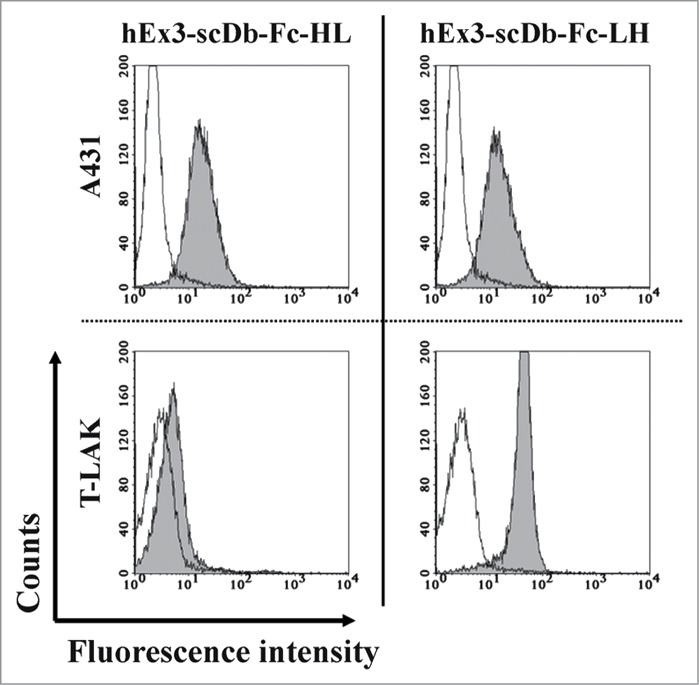
Confirmation of the cross-linking ability of hEx3-scDb-Fcs. A431 and T-LAK cells were incubated with PBS as a negative control (open area) or with each hEx3-scDb-Fc (shaded area); this incubation was followed by staining with FITC-CD3εγ for A431 cells (upper panels) or with FITC-sEGFR for T-LAK cells (lower panels).

We recently reported that cytotoxic enhancement of domain order–rearranged hEx3-LH has little correlation with its binding affinities, but could be attributed to its structural superiority for cross-linking.^18^ To compare the growth inhibitory effects and binding affinities of hEx3-scDb-Fc-HL and hEx3-scDb-Fc-LH, we performed kinetic analyses for immobilized sEGFR by using SPR measurements. A lower dissociation rate caused by the bivalency of hEx3-scDb-Fcs contributed to their higher affinity for sEGFR than those of hEx3-Dbs, hEx3-HL, and hEx3-LH, but no major differences between hEx3-scDb-Fc-HL and hEx3-scDb-Fc-LH were observed (**Table 1**). Because the binding kinetics for CD3 were not determined, due to the inactivation of immobilized CD3 on a sensor chip, we evaluated binding affinities for CD3 on the surface of T-LAK cells using flow cytometry; however, similar results were also found between hEx3-scDb-Fc-HL and hEx3-scDb-Fc-LH (**Fig. S1**). Then, to investigate which factors contributed to the increased growth inhibitory effects of hEx3-scDb-Fc-LH, we compared the cross-linking abilities of the hEx3-scDb-Fcs by using flow cytometry. We found that both molecules were able to cross-link between A431 cells and CD3εγ (**Fig. 4**, upper panel); however, only hEx3-scDb-Fc-LH showed effective cross-linking between T-LAK cells and sEGFR (**Fig. 4**, lower panel). The structure of hEx3-scDb-Fc-LH might be able to avoid steric hindrance with cell surface molecules, particularly those of T-LAK cells, and this feature might contribute to the increased cytotoxic effects of this bsAb, indicating that the characteristics of hEx3-LH are fully retained after Fc fusion.

**Table 1.** Binding and pharmacokinetic parameters

	*k*_on_ ( × 10^5^ M^−1^s^−1^)	*k*_off_ ( × 10^−3^ s^−1^)	*K*_A_ ( × 10^7^ M)	AUC (1.5–8)
hEx3-HL	3.7*^a^*	5.0 *^a^*	7.3 *^a^*	8.0
hEx3-LH	3.7 *^a^*	7.6 *^a^*	4.9 *^a^*	14.3
hEx3-scDb-Fc-HL	3.0	0.60	39.5	24.7
hEx3-scDb-Fc-LH	1.2	0.57	21.3	44.5
hEx3-scDb-Fc-LH-IgG2	1.5	0.18	84.0	n.d.*^b^*

Kinetic parameters were calculated by means of a global fitting analysis with the assumption of a 1:1 Langmuir binding model. ^a^Data from our previous report^18^; ^b^n.d., not determined.

### Effect of the domain order of hEx3-scDb-Fc on cytokine production

**Figure 5.**
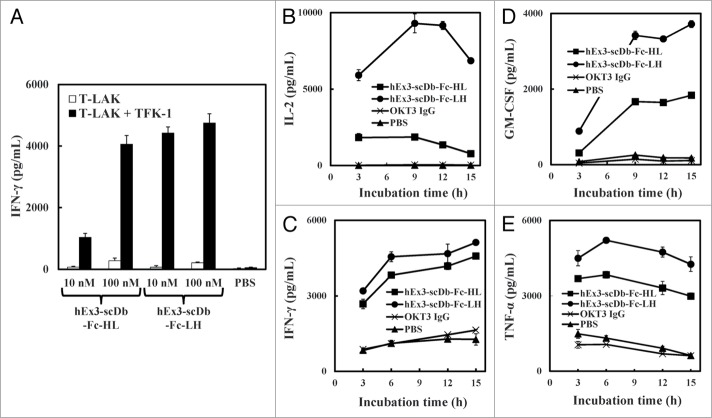
hEx3-scDb-Fcs-mediated cytokine production by T-LAK cells. IFN-γ concentration was evaluated by using an ELISA (**A**). Time courses of IL-2 (**B**), IFN-γ (**C**), GM-CSF (**D**), and TNF (**E**) production were evaluated by using an ELISA after 3–15 h of co-culturing 10 nM antibodies with T-LAK cells (1 × 10^5^) in the presence of overnight-adhered TFK-1 cells (5 × 10^3^).

To investigate whether the observed differences in cross-linking ability affect cytokine production by T-LAK cells, we analyzed the concentrations of interferon (IFN)-γ in the supernatant of T-LAK cells cultured with either hEx3-scDb-Fc construct in the presence or absence of TFK-1 cells. Both bsAbs induced IFN-γ production by T-LAK cells at a low level in the absence of target cells. In contrast, in the presence of target cells, hEx3-scDb-Fc-LH mediated substantial IFN-γ production; at concentrations of 10 nM, hEx3-scDb-Fc-LH resulted in a higher level of IFN-γ production than the HL type (**Fig. 5A**). At this concentration, although parental anti-CD3 IgG did not mediate any cytokine production, both bsAbs especially the LH-type mediated substantial production of IL-2, IFN-γ, GM-CSF, and TNF via cross-linking during the 3–15 h time course of co-culturing (Fig. 5BCDE). Thus, the structural superiority of the LH-domain order appeared to increase the cytokine production, resulting in the enhanced cancer growth inhibitory effects of hEx3-scDb-Fc-LH.

### Confirmation of the in vitro and in vivo stability of hEx3-scDb-Fcs

**Figure 6.**
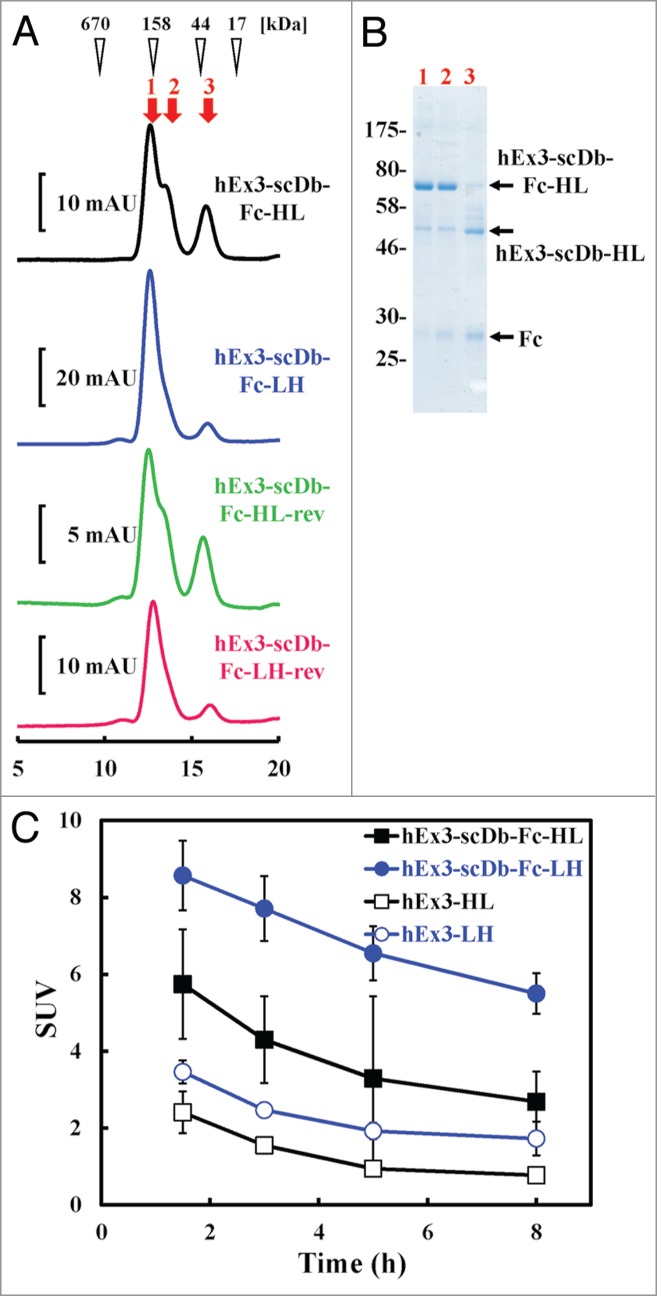
Stability assessment of hEx3-scDb-Fcs with different domain orders. (**A**) Gel filtration of hEx3-scDb-Fcs to assess their stability after storage. Fractionated hEx3-scDb-Fcs were stored for 3 wk at 4°C and then applied to a Hiload Superdex 200-pg column (10/300). (**B**) Reducing SDS-PAGE of each gel filtration fraction of hEx3-scDb-Fc-HL, indicated by the arrows in A. (**C**) Blood clearance of bsAbs. Imprinting control region (ICR) mice (n = 5) were injected with each of the ^125^I-labeled bsAbs, and blood samples were collected from tail veins at the indicated time points.

Structural stability is an essential factor for potential therapeutic proteins, particularly designed proteins. We evaluated the stability of the molecular structures of the hEx3-scDb-Fcs by use of gel filtration analysis using fractionated monomers that had been stored for 3 wk at 4°C. A shoulder on the major peak and an additional smaller peak were apparent on the chromatograph (**Fig. 6A**). SDS-PAGE analysis identified these fractions as hEx3-scDb-Fcs derivatives that had fragmented in the region of the connecting site of the Fc region during long-term storage; results for hEx3-scDb-HL are shown in **Figure 6B**as a representative example. Notably, the fragmentation rate of the LH-types appeared to be slower than that of the HL-types. To evaluate in vivo stability, we measured the area under the curve (AUC) using radioiodine-labeled bsAbs. The AUCs_(1.5–8 h)_ of hEx3-scDb-Fc-HL and hEx3-scDb-Fc-LH were 3.1-fold greater than those of their corresponding Db formats, hEx3-HL and hEx3-LH, as a result of the increased molecular weight due to the Fc fusion (**Fig. 6C**; **Table 1**). The AUCs observed for the LH-types were 1.8 times those of the HL types. These results suggest that the LH domain order has structural superiority not only in terms of cross-linking ability, but also with respect to in vitro and in vivo stability.

### Influence of the subtype of Fc region on the growth inhibition effects of scDb-Fcs

**Figure 7.**
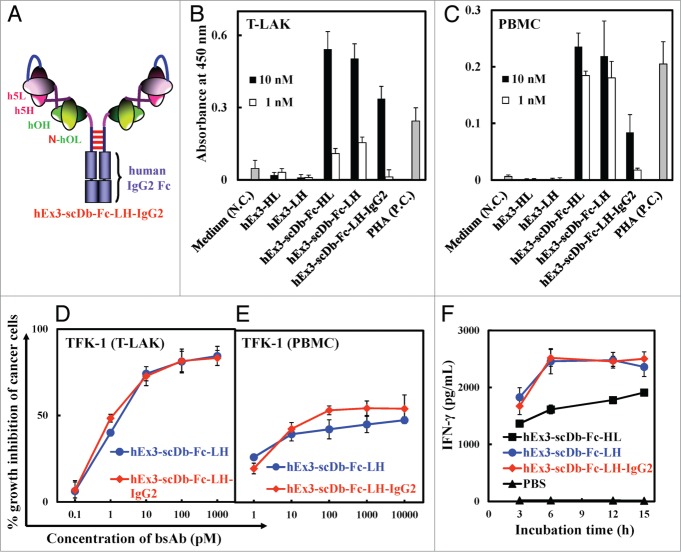
Comparison of bsAbs with hEx3-scDb-Fc-LH-IgG2. (**A**) Schematic diagram of hEx3-scDb-Fc-LH-IgG2. Proliferative effects of bsAbs on T-LAK cells (**B**) and PBMCs (**C**). Freshly isolated PBMCs or T-LAK cells were incubated for 72 h with the indicated doses of bsAbs. RPMI 1640 medium and phytohemagglutinin (PHA) served as the negative control (N.C.) and positive control (P.C.), respectively. To compare the growth inhibitory effects of bsAbs with that of hEx3-scDb-Fc-LH-IgG2, hEx3s and T-LAK cells (**D**) or PBMCs (**E**) were added to TFK-1 cells at ratios of 2:1 and 15:1, respectively. Data are presented as the mean ± 1 s.d. and are representative of at least two independent experiments. A time course of IFN-γ production was evaluated by using an ELISA after 3–15 h of co-culturing 10 nM bsAbs with T-LAK cells (1 × 10^5^) in the presence of overnight-adhered TFK-1 cells (5 × 10^3^) (**F**).

To investigate whether the ADCC activity contributed to the enhanced growth inhibition effects of scDb-Fc, we constructed an hEx3-scDb-Fc-LH variant whose Fc region was derived from human IgG2 (hEx3-scDb-Fc-LH-IgG2, **Figure 7A**). Reduced proliferative effects were observed with the IgG2 subtype compared with the IgG1 subtype, especially for PBMCs, because the population of Fcγ receptor III–positive lymphocytes in PBMCs is larger than that in T-LAK cells (Fig. 7BC). However, no major differences between the IgG1 subtype and IgG2 subtype were observed with respect to affinity (**Table 1**), growth inhibitory effect (even with PBMCs as effector cells; Figure 7DE), or cytokine production (**Fig. 7F**). These findings suggest that the contribution of the ADCC activity to the growth inhibition effects of hEx3-scDb-Fc-LH may be minimal in vitro.

### Comparison of the in vivo anti-tumor effects of hEx3-scDb-Fc-LH

**Figure 8.**
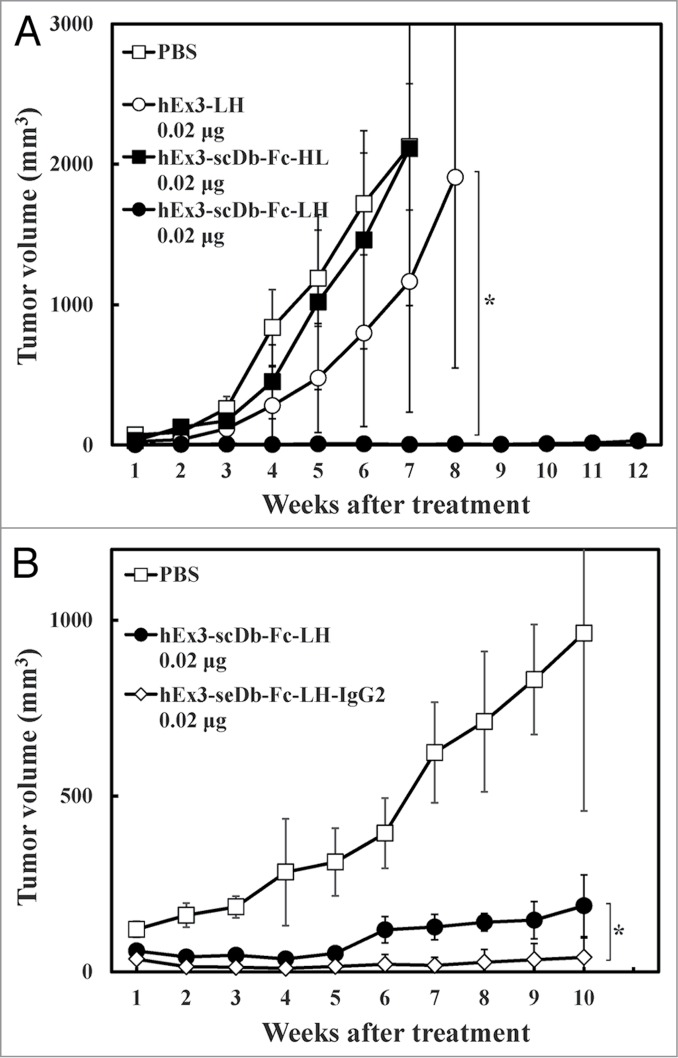
In vivo antitumor effect of hEx3-scDb-Fc-LH. Points indicate the mean tumor volumes from each treatment group; bar, ± 1 s.d.; *, Significant (*p* < 0.05) difference between hEx3-scDb-Fc-LH and hEx3-LH (**A**) or hEx3-scDb-Fc-LH-IgG2 (**B**).

To evaluate the influence of the domain order or structural format of hEx3-scDb-Fc on the in vivo anti-tumor effects of the reagent, we transplanted mixtures of TFK-1 cells and T-LAK cells into SCID mice, which we then treated for 4 d with various samples. Although 0.02 μg of hEx3-scDb-Fc-HL and hEx3-LH without the Fc region produced no substantial differences compared with the PBS control, the same dose of hEx3-scDb-Fc-LH significantly inhibited tumor growth in the SCID mice (**Fig. 8A**). Further, these tumor-inhibitory effects were enhanced with the IgG2 variant of hEx3-scDb-Fc-LH compared with the IgG1 subtype, hEx3-scDb-Fc-LH (**Fig. 8B**). Enhanced anti-tumor effects, to which ADCC activity contributed little, were also found this in vivo therapeutic model by rearranging the domain order of hEx3-scDb-Fc.

## Discussion

To develop more beneficial therapeutic antibodies, studies have examined the effects of configuration and antibody-fragment type on the function of antibodies, especially scFv-based IgG-like bsAbs.^11,12,19^ Although there have been no detailed studies to date examining the influence of the domain order of the variable fragments on the quality of IgG-like bsAbs, the important roles of linker and domain orientation on the function of dual variable domain immunoglobulin (DVD-Ig^TM^) proteins, one of the IgG-like formats with tetravalency, have recently been reported.^20^ In the present study, we prepared four types of domain-rearranged Db-based IgG-like bsAbs to examine the influence of the domain order of a bsDb on the function of its Fc-fusion format. Similar chromatographs were observed for all purified hEx3-scDb-Fcs (**Fig. 1C**); however, hEx3-scDb-Fcs with the VL–VH order (hEx3-scDb-Fc-LHs) inhibited tumor cell growth more effectively than did those with the VH-VL order (hEx3-scDb-Fc-HLs) (**Fig. 2A**). Together with our recent report that the VL–VH order is the most effective domain configuration for hEx3-Dbs,^18^ these results indicate that an effective domain order of bsDbs can be retained after Fc fusion and suggest that Db-based IgG-like bsAbs may be markedly improved by optimizing their constitutive Dbs.

A previous study showed that increasing the binding affinity of bsAbs through mutagenesis can enhance their cytotoxicity.^21^ Therefore, when two bsAb share an identical format, their respective affinities may correlate with their efficacy. However, as we found with hEx3-Dbs,^18^ the enhanced cytotoxicity of hEx3-scDb-Fc-LH was correlated with structural differences in cross-linking between target cells (**Fig. 4**, lower panel), but not with differences in binding affinities (**Table 1**). In contrast, the manner in which hEx3-Db was fused to the Fc region, i.e., the relative position of the bsDb against the Fc region, did not affect the growth inhibition effects (**Figs. 2A and 3C**). These results suggest that it is important for a bsDb-Fc to have a bsDb that avoids steric hindrance with molecules near the target antigens in order to induce effective tumor growth inhibition, but steric hindrance with the Fc region must also have no appreciable effects.

An upper hinge in IgG1 has been shown to be vulnerable to various degradation mechanisms, such as papain cleavage, β-elimination reactions, and radical-mediated reactions.^22,23^ Cleavage at this upper hinge was also found in all of our hEx3-scDb-Fcs; however, hEx3-scDb-Fc-LHs showed greater resistance to degradation compared with -HLs (**Fig. 6A**). Further, the AUCs_(1.5–8 h)_ of the hEx3-Dbs were increased by Fc fusion, and also by conversion to the VL-VH order, which resulted in hEx3-scDb-Fc-LH having the largest AUCs_(1.5–8 h)_, comparable to that of cetuximab (40.5 h), under the same conditions (**Fig. 6C**; **Table 1**). Different half-lives have also been reported for scFv-based IgG-like bsAbs depending on their configurations.^11,12^ Although the reasons for these differences are unknown, our results show that merely rearranging the domain order of scDb can produce a scDb-Fc with not only higher activity, but also better in vitro and in vivo stability.

IgG-like bsAbs are attractive formats because they usually show multivalent binding, have prolonged half-lives, can be purified with protein A, and induce Fc-mediated effector functions;^9,10^ however, these functions, in addition to cross-linking effects, can cause severe side effects, such as the induction of a cytokine storm. The IgG2 subtype of hEx3-scDb-Fc-LH showed comparable in vitro and in vivo functions to those of the IgG1 subtype, with the exception of its proliferative effects on PBMCs (**Figs. 7** **and** **8**; **Table 1**), suggesting that Fc-mediated effector functions such as ADCC are not essential for the induction of the effective anti-tumor activity of hEx3-scDb-Fc-LH. Although IgG-like bsAbs have been difficult to express in bacteria due to their large size, several recent reports have shown the successful preparation of full-length IgG and also IgG-like bsAbs in *E. coli*.^24,25^ Glycosylation, which is indispensable for induction of ADCC but not for a long half-life,^26,27^ does not occur during the preparation of Fc-fusion proteins in bacteria, which is clearly a disadvantage. However, bacterial expression of hEx3-scDb-Fc-LH, which need not induce ADCC, may be an ideal approach to produce this beneficial therapeutic antibody at a low production cost.

Another drawback of classic bsAbs prepared through chemical conjugation or quadroma production is their reduced affinity due to monovalency to each antigen, unlike conventional IgGs. Although several IgG-like bsAb formats with tetravalency, for example, bivalent bivalency,^1,10,28^ have been designed, so far none have been approved as drugs. Further, use of bsAbs recruiting T cells via CD3 may have some undesirable effects. For example, one of the most advanced bsAbs targeting CD3, blinatumomab, has shown promising results in a clinical study, but, in some cases, patients receiving the drug experienced central nervous system symptoms that have led to permanent discontinuation of study drug.^29^ Therefore, at present, it is unclear which IgG-like bsAb format and which strategy are the best; however, in addition to examining configurations and kinds of antibody, rearranging the domain order of IgG-like bsAbs, including scFv-based bsAbs, may be an ideal strategy to optimize or improve the development of these clinical reagents.

## Materials and Methods

### Construction of expression vectors for hEx3-scDb-Fcs with different domain orders

As in our previous report,^13^ we describe here the VH and VL regions of the humanized anti-EGFR antibody 528 as h5H and h5L and those of the humanized anti-CD3 antibody OKT3 as hOH and hOL. We previously described the construction of the mammalian expression vector pcDNA-hEx3-scDb-3C-Fc for hEx3-scDb-3C-Fc, in which a single-chain form of hEx3-Db was fused to a human IgG1 Fc region via a recognition site (LEVLFQGP) for human rhinovirus 3C (HRV3C) protease.^30^ Because two chimeric single-chain components of hEx3-Db—hOHh5L and h5HhOL, both with a VH–VL order—were connected via a 20–amino acid linker ([GGGGS]4) in the hOHh5L–h5HhOL order, hEx3-scDb-3C-Fc was re-designated as hEx3-scDb-Fc-HL in this report for clarity. The expression vector pcDNA-hEx3-scDb-Fc-LH for hEx3-scDb-Fc-LH with a VL–VH order (i.e., a hOLh5H–h5LhOH–3C–Fc order), was constructed by using the overlap polymerase chain reaction. The remaining vectors were similarly constructed: pcDNA-hEx3-scDb-Fc-HL-rev for hEx3-scDb-Fc-HL-rev with a h5HhOL–hOHh5L order, in which the scDb has the VH–VL order, but the relative position of each variable region is the reverse of hEx3-scDb-Fc-HL; pcDNA-hEx3-scDb-Fc-LH-rev for hEx3-scDb-Fc-LH-rev with a h5LhOH–hOLh5H order, in which the scDb has the VL–VH order, but the relative position of each variable region is the reverse of hEx3-scDb-Fc-LH (**Fig. 1B**). The leader peptide sequences for protein secretion were derived from the mouse OKT3 heavy chain for the HL-types and the light chain for the LH-types, respectively.^31^ To construct the expression vector pcDNA-hEx3-scDb-Fc-LH-IgG2 for hEx3-scDb-Fc-LH-IgG2, the gene for the human Fc region derived from the IgG2 subclass was cloned from peripheral blood mononuclear cells (PBMC) and then used to replace the IgG1 Fc portion in pcDNA-hEx3-scDb-Fc-LH.

### Preparation of bsDbs

The methods for expression using CHO cells and purification have been described previously.^30^ In brief, IgG-like bsAbs were first purified on a protein A column (GE Healthcare Bio-Science Corp., Piscataway, NJ, USA), and gel filtration analysis with a Hiload Superdex 200-pg column (26/60; GE Healthcare) was then used to fractionate the monomers of each bsAb. The column was equilibrated with phosphate-buffered saline (PBS), and 5 mL of purified bsAb was then loaded onto the column at a flow rate of 2.5 mL/min. The long-term stability of bsAbs in storage was evaluated by using a Hiload Superdex 200-pg column (10/300; GE Healthcare). The column was equilibrated with PBS, and then 0.25 mL of purified bsAb was loaded onto the column at a flow rate of 0.5 mL/min. To prepare each scDb, IgG-like bsAbs were digested by HRV3C protease fused to glutathione S-transferase (PreScission protease; GE Healthcare) according to the protocol described by the manufacturer.^32^ The protease was removed on a Glutathione Sepharose 4B column (GE Healthcare), and the flow-through was loaded on to the protein A column again to remove the digested Fc and undigested Fc fusion protein. The presence and purity of the bsAbs and fragments were confirmed by sodium dodecyl sulfate-PAGE (SDS-PAGE) at each stage of purification. Both hEx3-HL and hEx3-LH without the Fc region were prepared by using the bacterial expression system described previously.^18^

### Cell lines

Human bile duct carcinoma (TFK-1), human epidermoid cancer (A431), and human breast cancer (MCF-7) cell lines were used in this study. The TFK-1 cell line was established in our laboratory,^33^ A431 and MCF-7 were obtained from the Cell Resource Center for Biomedical Research, Institute of Development, Aging and Cancer, Tohoku University (Sendai, Japan). These cell lines were cultured with RPMI 1640 medium supplemented with 10% fetal bovine serum (FBS), 100 U/mL penicillin, and 100 μg/mL streptomycin.

### In vitro killing assay

Lymphokine-activated killer cells with the T-cell phenotype (T-LAK) were induced as previously described.^34^ In brief, PBMC were cultured for 48 h at a density of 1 × 10^6^ cells/mL in medium supplemented with 100 IU/mL of recombinant human IL-2 (Shionogi Pharmaceutical Co., Osaka, Japan) in a culture flask (A/S Nunc, Roskilde, Denmark) that was precoated with anti-CD3 monoclonal antibody (10 μg/mL).

The in vitro growth inhibition of the cancer cells was assayed with a 3-(4,5-dimethylthiazole-2-yl)-5-(3-carboxymethoxyphenyl)-2-(4-sulfophenyl)-2*H*-tetrazolium inner salt (MTS) assay kit (CellTiter 96 AQueous Non-Radioactive Cell Proliferation Assay; Promega) as reported previously.^34^

### Surface plasmon resonance spectroscopy

The interactions between soluble EGFR (sEGFR) and bsAbs were analyzed by using surface plasmon resonance (SPR) spectroscopy (Biacore 2000, GE Healthcare). The methods for the expression and purification of sEGFR have been described previously.^35^ sEGFR was immobilized on a sensor chip CM5 up to 3690 resonance units. Various concentrations of bsAbs in PBS with 0.005% Tween 20 were allowed to flow over the bound sEGFR at a flow rate of 20 μL/min at 25°C. The surface was regenerated with 10 mM Glycine–HCl (pH 2.0) with no loss of activity. The data were referenced by subtracting the response of a blocked blank cell. BIAevaluation software (GE Healthcare) was used to analyze the data. Kinetic parameters were calculated by means of a global fitting analysis with the assumption of a 1:1 Langmuir binding model.

### Confirmation of cross-linking ability

Fluorescein isothiocyanate-labeled CD3εγ (FITC-CD3εγ) and sEGFR (FITC-sEGFR) were prepared by using Fluorescein Labeling Kit-NH2 (Dojindo Laboratories, Kumamoto, Japan) to confirm cross-linking between CD3εγ and A431 cells and between sEGFR and T-LAK cells, respectively. The methods for the expression and purification of CD3εγ have been described previously.^36^ Target cells (1 × 10^6^) were incubated for 30 min on ice with 100 pmol of each bsDb. After a wash with PBS containing 0.1% NaN_3_, the cells were exposed for 30 min to 1 μg of FITC-CD3εγ or FITC-sEGFR on ice. Stained cells were analyzed by use of flow cytometry (FACSCalibur, Becton Dickinson).^18^

### Enzyme-linked immunosorbent assay

Each bsAb was co-cultured with T-LAK cells (5 × 10^4^) in the presence or absence of overnight-adhered TFK-1 cells (5 × 10^3^). After 16 h of co-culture, supernatants were harvested and used in an enzyme-linked immunosorbent assay (ELISA) for IFN-γ (ELISA Ready-SET-Go!, Bay Bioscience, Hyogo, Japan) according to the manufacturer's instructions. To evaluate the time course of cytokine production, each bsAb or OKT3 IgG at a final concentration of 10 nM was co-cultured with T-LAK cells (1 × 10^5^) in the presence of overnight-adhered TFK-1 cells (5 × 10^3^). After 3–15 h of co-culture, the supernatants were harvested and subjected to the ELISAs for human IL-2, IFN-γ, GM-CSF, and TNF (Bay Bioscience).

### Radiolabeling of bsAbs

An iodogen tube was prepared by coating a microfuge tube (1.5 mL) with iodogen (100 μg/tube, Thermo Fisher Scientific, Waltham, MA, USA) in-house; the tube was then used to radiolabel bsAbs with [^125^I]NaI (74 MBq/0.1 mL, Perkin–Elmer). Each bsAb (600–900 μL, 89–108 μg), fractionated monomer using gel filtration, was placed in an iodogen tube with [^125^I]NaI (20–25 μL, 26–37 MBq) and incubated for 15 min at room temperature with Vortex mixing. The ^125^I-labeled bsAb was then separated from the unreacted [^125^I]NaI by using size-exclusion chromatography with a Bio-Gel P-6 Desalting Cartridge (10 mL, BIO RAD), eluting with PBS-Tween 20 (0.05%) at a flow rate of 1.5 mL/min. The radiochemical purities of the isolated bsAbs ranged from 94%– 98%.

### Blood clearance study

The Ethics Committee for Experimental Research in Animals of Tohoku University approved the study protocol. Male imprinting control region (ICR) mice (6-wk-old, 27–31 g) were injected in the lateral tail vein with the ^125^I-labeled bsAb (2 μg, 150–180 kBq) in a PBS solution (0.2 mL). A portion of blood (∼10 μL) was collected from the contralateral tail vein at 1.5, 3, 5, and 8 h post-injection (n = 5 at each time point). Radioactivity and weight of the blood were measured with a gamma counter (AccuFLEXγ7000; Hitachi Aloka Medical). Blood radioactivity was expressed as the standardized uptake value (SUV), which was defined as follows: SUV = (Blood radioactivity / Blood weight) / (Injected radioactivity / Body weight).

### Proliferation assay

Proliferation of PBMCs and T-LAK cells was assessed in a Cell Proliferation ELISA system (GE Healthcare) as described previously.^14^ Briefly, 1 × 10^5^ freshly isolated PBMCs or T-LAK cells were distributed into each well of 96-well flat-bottomed plates (Sumitomo Bakelite Ltd.) in the presence of various concentrations of bsAbs. After incubation of the plates for 48 h at 37°C, 5-bromo-2′-deoxyuridine (BrdU) labeling reagent was added and the cells were incubated for an additional 24 h.

### In vivo tumor models

For each mouse, 1.0 × 10^7^ T-LAK cells were mixed with 5 × 10^6^ TFK-1 cells in a final volume of 0.15 mL of PBS. The mixture was then injected subcutaneously into the dorsal thoracic wall of female, 6-wk-old severe combined immunodeficient (SCID) mice (CLEA Japan). Then, five mice per group were treated intravenously with bsAb or PBS at the indicated doses starting at 1 h after TFK-1 inoculation, and treatment was repeated once daily for 3 consecutive days. Tumor size was measured weekly by using a caliper, and the approximate tumor volume (V, in mm^3^) was calculated from linear measurements of the width (A, in mm) and length (B, in mm) as follows: V = (A^2^ × B)/2. Experiments involving mice were reviewed by the Committee on Ethics in Animal Experiments of Tohoku University and were performed under the Guidelines for Animal Experiments of Tohoku University and according to the laws and notifications of the Japanese government.
