# Supplementary material for: Rearranging the domain order of a diabody-based IgG-like bispecific antibody enhances its antitumor activity and improves its degradation resistance and pharmacokinetics
Source: MAbs. 2014 Oct 30;6(5):1243–54. doi: 10.4161/mabs.29445 (PMC4623410; doi:10.4161/mabs.29445)
Supplement: 2014MABS0580R-Sup.pdf [file kmab-06-05-972878-s001.pdf]

Landes Bioscience
www.landesbioscience.com

Supplemental Material to:

Ryutaro Asano, Ippei Shimomura, Shota Konno, Akiko Ito, Yosuke Masakari, Ryota Orimo, Shintaro Taki, Kyoko Arai, Hiromi Ogata, Mai Okada, Shozo Furumoto, Masayoshi Onitsuka, Takeshi Omasa, Hiroki Hayashi, Yu Katayose, Michiaki Unno, Toshio Kudo, Mitsuo Umetsu, and Izumi Kumagai

Rearranging the domain order of a diabody-based IgG-like bispecific antibody enhances its antitumor activity and improves its degradation resistance and pharmacokinetics

mAbs 2014; 6(5)
http://dx.doi.org/10.4161/mabs.29445

http://www.landesbioscience.com/journals/mabs/article/29445/

Supplementary Figure 1

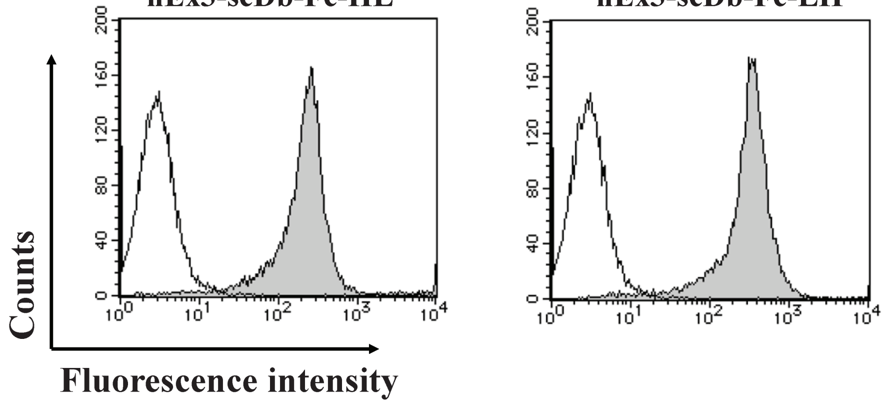

Flow cytometric analysis of reactivity of each hEx3-scDb-Fc with T-LAK cells. T-LAK cells were incubated with PBS as a negative control (open area) or with each hEx3-scDb-Fc (shaded area); this incubation was followed by staining with FITC-conjugated anti-human IgG antibody.
